# Clinicopathological and prognostic study of idiopathic membranous nephropathy with tip or non-tip variant focal segmental glomerulosclerosis: a single-center cohort study

**DOI:** 10.1080/0886022X.2025.2512052

**Published:** 2025-06-08

**Authors:** Shungang Xu, Xueting Liu, Xuanwen Chen, Bo Chen, Sishi Lin, Xiaohan You, Duo Li, Ji Zhang, Chaosheng Chen

**Affiliations:** ^a^ Department of Nephrology, The First Affiliated Hospital of Wenzhou Medical University, Wenzhou, Zhejiang, PR China; ^b^ Institute of Chronic Kidney Disease, Wenzhou Medical University, Wenzhou, Zhejiang, PR China

**Keywords:** Idiopathic membranous nephropathy, focal segmental glomerulosclerosis, tip lesion variant, chronic kidney disease

## Abstract

Idiopathic membranous nephropathy (IMN) complicated with focal segmental glomerulosclerosis (FSGS) is not rare, but the impact of variant pathological manifestations of FSGS (tip variant vs non-tip variant) on the clinical and prognosis of IMN patients remains to be further studied. A total of 536 eligible IMN patients were enrolled in the study and divided into three groups based on the variant histopathological presence of FSGS: 387 patients without FSGS (IMN group), 67 patients with tip variant FSGS (tpFSGS group), and 82 patients with non-tip variant FSGS (ntpFSGS group). IMN patients with FSGS had significantly lower serum albumin (IMN vs. tpFSGS vs. ntpFSGS, 21.20 [17.15, 25.25] vs. 18.60 [16.05, 22.10] vs. 17.90 [15.40, 22.03], *p* < 0.001) and higher 24-hour proteinuria (5.65 [3.60, 7.69] vs. 6.11 [3.97, 8.46] vs. 6.98 [4.77, 8.49], *p =* 0.003). Additionally, the ntpFSGS group exhibited more advanced histopathological manifestations (pathological stage, *p <* 0.05). Otherwise, a lower complete remission rate was observed in the ntpFSGS group. Furthermore, multivariate Cox regression analysis demonstrated significantly lower remission in tpFSGS [HR = 0.69, *p =* 0.029] and ntpFSGS [HR = 0.66, *p =* 0.014] groups. Adjusted by the immunosuppressive therapy, a worse prognosis was observed in the ntpFSGS group [KM curve, log-rank, *p <* 0.05], but no significant difference in the IMN group and the tpFSGS group [KM curve, log-rank, *p =* 0.74]. Our cohort study suggests that idiopathic membranous nephropathy patients with secondary variant FSGS indicated different clinical significance.

## Introduction

Idiopathic membranous nephropathy (IMN) is one of the most common causes of nephrotic syndrome in adults [1]. The natural course of IMN is variable, with spontaneous remission in approximately 1/3 of patients, persistent proteinuria in another 1/3, and gradual progression to end-stage renal disease (ESRD) in the remaining 1/3 [2,3]. Increased serum creatinine levels, persistent heavy proteinuria, high serum titers of anti-M-type phospholipase A2 receptor (anti-PLA2R) antibody, and existence of tubular atrophy and interstitial fibrosis had been reported as poor prognostic indicators for progression to ESKD in IMN patients [4–7].

The term focal segmental glomerulosclerosis (FSGS) has been applied to segmental sclerosing lesions in different circumstances, such as nephrotic syndrome, non-nephrotic proteinuria, states of reduced renal mass, and various glomerular disorders [8]. Thirty years ago, Lee and Koh conducted a retrospective study on the clinicopathological characteristics of patients with and without FSGS [9]. The prognostic effect of FSGS on IMN remains controversial. Previous studies revealed that IMN patients with FSGS lesions had increased blood pressure, decreased serum albumin, and increased severity of tubulointerstitial and vascular lesions [10–12]. Moreover, prior studies revealed that the coexistence of focal segmental glomerulosclerosis poses a significant risk for persistent proteinuria in patients with IMN [10,12,13]. Furthermore, it independently contributes to the deterioration of renal function [9,12,14]. Nevertheless, conflicting findings have been reported in other studies [11,15].

Few previous studies have specifically addressed the different variants of FSGS in relation to IMN. He et al. explored the clinical features and prognosis of IMN combined with atypical FSGS [13]. Wang et al. analyzed the differences in clinical features and prognoses between IMN combined with tip variant FSGS and non-tip variant FSGS, but the sample sizes of the studies were small, and the follow-up time was short [10]. In this study, we aimed to observe the clinicopathological characteristics of IMN combined with either tip variant or non-tip variant FSGS using a larger sample size, thereby providing valuable insights for clinical diagnosis and treatment.

## Methods

### Patient information

There were a total of 1368 cases of membranous nephropathy diagnosed by renal biopsy in our hospital from January 2010 to May 2022, and 536 patients with IMN were ultimately included according to the following inclusion and exclusion criteria. The inclusion criteria were as follows: the patient was diagnosed with membranous nephropathy by renal biopsy. The exclusion criteria were as follows: (1) membranous nephropathy was attributed to a secondary etiology, including autoimmune diseases, infectious diseases, cancer, hematological malignancies, drugs, and toxicant; (2) renal pathology revealed the presence of concurrent diabetic nephropathy; (3) renal pathology revealed acute tubulointerstitial injury; (4) the baseline clinical parameters are missing, including serum creatinine, urine protein, serum albumin, and others; however, the integrity of Anti-PLA2R data is not required; (5) patients with a follow-up duration of less than 3 months; or (6) the number of glomeruli in the renal puncture specimen is less than 10. A total of 536 patients who met the above criteria were divided into 3 groups according to the presence or absence of FSGS in renal biopsy tissues and the different FSGS variants. There were 387 patients without FSGS (IMN group), 67 patients with tip variant FSGS (tpFSGS group), and 82 patients with non-tip variant FSGS (ntpFSGS group). The study was approved by the Ethics Committee of the First Affiliated Hospital of Wenzhou Medical University.

### Clinical parameters and laboratory data

We collected clinical parameters and laboratory data, including sex, age, blood pressure (mm Hg), hemoglobin (g/L), serum creatinine (s-Cr, mg/dL), serum albumin (g/L), uric acid (mg/dL), blood urea, total cholesterol (mmol/L), triglycerides (mmol/L), high-density lipoprotein (mmol/L), low-density lipoprotein (mmol/L), serum fibrinogen (g/L), 24-h proteinuria (g/d), the urine protein-to-creatinine ratio (mg/g), and serum anti-PLA2R antibodies, from the enrolled patients at the time of the first renal biopsy. The eGFR was calculated using the modified eGFR-EPI formula [16].

### Renal biopsy

**Figure 1.**
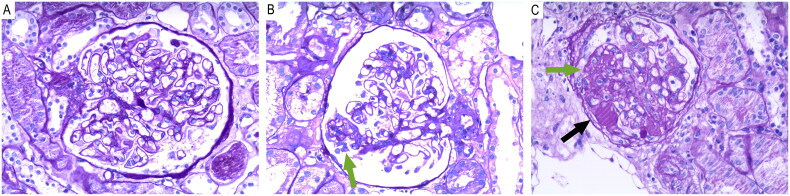
The light microscopic patterns of the lesions of the three groups (PAS; magnification: 200×). (a) IMN without FSGS; (b) IMN with tip variant FSGS; (c) IMN with non-tip variant FSGS. Green arrow indicates the histopathological features of segmental glomerulosclerosis. Black arrow indicates the histopathological features of segmental hyalinosis.

All the samples were stained with hematoxylin-eosin, periodic acid-Schiff, periodic acid-cresolamine silver, and elastic-Mason trichrome for light microscopy (LM), and the frozen samples were subjected to direct immunofluorescence (IF). Glomerular membranous lesions were categorized into 4 stages according to Ehrenreich–Churg staging [17]. Stage III and IV collapse into one group due to limited cases. On the basis of the LM results, statistics were generated on the number of glomeruli, global sclerosis, mesangial hypercellularity, tubular atrophy, interstitial fibrosis, arteriolar hyalinosis, and interstitial inflammatory cell infiltration. FSGS with tip lesion is defined by the presence of at least one segmental lesion involving the tip region, with either adhesion between the glomerular tuft and Bowman’s capsule at the tubular lumen or neck, or confluence of podocytes with parietal or tubular epithelial cells at the tubular lumen or neck, in the absence of perihilar or collapsing lesions (shown in Figure 1). FSGS with non-tip lesion includes collapsing, cellular, perihilar, and nonspecific (NOS) FSGS. In our study, tubulointerstitial lesions were defined as tubular atrophy or interstitial fibrosis involving more than 25% of the renal tissue. Acute tubulointerstitial lesion is defined by the following pathological features: damage to the renal tubular epithelium, including cellular edema, degeneration, detachment, and necrosis; infiltration of inflammatory cells (neutrophils, monocytes, lymphocytes); formation of casts within the tubular lumen; and interstitial edema. Acute tubulointerstitial lesion is defined as the presence of these lesions in more than 30% of the tubules and interstitium. Mesangial hypercellularity was defined as an increase in the number of cells within the mesangial region of the glomerulus. Benign arteriolar nephrosclerosis was characterized by the thickening and hyalinization of the walls of small renal arteries and arterioles. Arteriolar hyalinosis was defined as the accumulation of homogeneous, eosinophilic hyaline material within the walls of arterioles. Inflammatory cell infiltration was defined as the presence of immune cells, such as lymphocytes, macrophages, and neutrophils, within the renal tissue. On the basis of the IF results, statistics were generated on the deposition sites of immunoglobulin (IgG, IgA, and IgM), complement (C3, C4, and C1q), and fibrinogen.

### Endpoint events

The endpoint event was defined as remission of disease, which encompassed both complete remission (CR) and partial remission (PR). CR was defined as a urine protein-to-creatinine ratio of less than 300 mg/g accompanied by a stable eGFR and serum albumin levels exceeding 35 g/L. PR was defined as a urine protein-to-creatinine ratio less than 3500 mg/g and a reduction of over 50% from baseline, in addition to a stable eGFR.

### Statistical analysis

The numerical data are presented as the means (SDs) or medians (interquartile ranges), and differences between groups were evaluated using variance analysis or the Kruskal–Wallis rank test. The categorical data are presented as counts with percentages (%), and differences between groups were analyzed using Pearson’s χ^2^ test. The Benjamini–Hochberg method was used for correction of multiple comparisons. Univariate and multivariate Cox regression models were constructed and optimized using forward-backward stepwise methods and the Akaike information criterion. The treatment plans were divided into the following groups as a categorical variable: (1) RASI: Patients only use angiotensin-converting enzyme inhibitor (ACEI) or angiotensin receptor blocker (ARB) within 2 years of onset; (This group as a control variable); (2) CNI ± GCs: On the basis of RASI, patients use calcineurin inhibitors (CNI) in combination with glucocorticoids (GCs) or use calcineurin inhibitors alone within 2 years of onset; (3) CTX+GCs: On the basis of RASI, patients use cyclophosphamide (CTX) in combination with Glucocorticoids within 2 years of onset; (4) Other treatment plans: Medication plans other than the above situations, including glucocorticoids alone, other immunosuppressants, and combined use of multiple immunosuppressants. The Kaplan–Meier curve was used to display the end point. All reported *p*-values were two-tailed, and *p <* 0.05 was considered statistically significant. R version 4.0.4 and R packages were used to perform the analyses and plots [18].

## Results

### Clinical parameters

According to the inclusion and exclusion criteria mentioned previously, a total of 536 patients with IMN were enrolled from patients who underwent renal biopsy at our hospital from January 2010 to May 2022. The median age of the patients at diagnosis was 53 years (range from 17 − 84). The male-to-female ratio was 1.46:1. Table 1 shows the differences between the IMN group, tpFSGS group, and ntpFSGS group. There were no significant differences in age or sex among the three groups. Compared with the IMN group, the tpFSGS group had significantly lower albumin levels (21.20 [17.15, 25.25] vs. 18.60 [16.05, 22.10], *p <* 0.001), lower HDL cholesterol levels (1.22 [1.01, 1.47] vs. 1.06 [0.82, 1.31], *p <* 0.001), higher 24-h proteinuria levels (5.65 [3.60, 7.69] vs. 6.11 [3.97, 8.46], *p <* 0.05), higher triglyceride levels (2.14 [1.61, 3.24] vs. 2.49 [1.81, 3.84], *p <* 0.05), higher serum creatinine levels (66.00 [53.00, 77.00] vs. 68.00 [60.00, 77.50], *p <* 0.05), and higher positive rates of serum anti-PLA2R antibodies (69.9% vs. 90.0%, *p <* 0.001). Similarly, compared with the IMN group, the ntpFSGS group had significantly lower albumin levels (21.20 [17.15, 25.25] vs. 17.90 [15.40, 22.03], *p <* 0.001), lower HDL cholesterol levels (1.22 [1.01, 1.47] vs. 1.10 [0.97, 1.34], *p <* 0.001), lower eGFRs (105.96 [97.94, 113.70] vs. 101.71 [92.59, 111.94]), higher 24-h proteinuria levels (5.65 [3.60, 7.69] vs. 6.98 [4.77, 8.49], *p <* 0.05), higher total cholesterol levels (7.25 [5.89, 8.79] vs. 8.40 [6.43, 10.35], *p <* 0.05), higher LDL cholesterol levels (4.19 [3.11, 5.42] vs. 4.92 [3.62, 6.87], *p <* 0.05), higher serum creatinine levels (66.00 [53.00, 77.00] vs. 68.00 [57.00, 85.00], *p <* 0.05), higher serum fibrinogen (4.59 [3.87,5.67] vs. 5.20[4.49, 6.01], *p <* 0.05), and higher positive rate of serum anti-PLA2R antibody (69.9% vs. 90.6%, *p <* 0.001). In addition, the disease course before renal biopsy in the ntpFSGS group was the longest among the three groups (3.5 [1.0, 12.0] vs. 2.0 [1.0, 12.0] vs. 6.0 [2.0, 12.0], *p <* 0.05). There were no significant differences in clinical parameters between the tpFSGS group and the ntpFSGS group.

**Table 1.** Comparison of the clinical manifestations among three groups at the time of renal biopsy.

	IMN group	tpFSGS group	ntpFSGS group	*p*
n	387	67	82	
Female, n (%)	162 (41.9)	23 (34.3)	33 (40.2)	0.508
Age (year), median [IQR]	52.00 [43.00, 60.00]	54.00 [43.50, 61.50]	52.50 [45.00, 64.00]	0.541
eGFR (mL/min/1.73 m^2^), median [IQR]	105.96 [97.94, 113.70]^‡^	103.28 [96.10, 112.13]	101.71 [92.59, 111.94]	0.053
MBP (mmHg), median [IQR]	113.33 [103.17, 121.00]	115.33 [108.42, 123.75]	115.33 [103.00, 129.67]	0.276
Serum creatinine (umol/L), median [IQR]	66.00 [53.00, 77.00]^‡^	68.00 [60.00, 77.50]^†^	68.00 [57.00, 85.00]	0.055
Proteinuria (g/24 h), median [IQR]	5.65 [3.60, 7.69]^‡^	6.11 [3.97, 8.46]^†^	6.98 [4.77, 8.49]	0.003
Serum albumin (g/L), median [IQR]	21.20 [17.15, 25.25]^‡^	18.60 [16.05, 22.10]^†^	17.90 [15.40, 22.03]	<0.001
TC (mmol/L), median [IQR]	7.25 [5.89, 8.79]^‡^	8.00 [6.61, 9.78]	8.40 [6.43, 10.35]	0.001
TG (mmol/L), median [IQR]	2.14 [1.61, 3.24]	2.49 [1.81, 3.84]^†^	2.60 [1.83, 3.45]	0.016
Serum uric acid (mmol/L), median [IQR]	365.00 [310.00, 426.50]	358.00 [307.00, 418.50]	371.00 [325.00, 432.00]	0.454
Hemoglobin (g/L), median [IQR]	133.00 [123.00, 144.00]	134.00 [119.50, 148.50]	129.00 [117.00, 142.00]	0.327
Serum fibrinogen (g/L), median [IQR]	4.59 [3.87, 5.67]^‡^	4.81 [4.24, 5.71]	5.20 [4.49, 6.01]	0.003
Blood urea (mmol/L), median [IQR]	4.60 [3.70, 5.80]	4.90 [3.70, 6.15]	4.80 [3.90, 6.10]	0.291
Anti-PLA2R positive, n (%)	151/216 (69.9)^‡^	45/50 (90.0)^†^	29/32 (90.6)	0.001
Follow-up (month),	42.0 [25.0, 68.5]^‡^	26.0 [9.5, 40.0]^†^	22.5 [4.0, 56.25]	<0.001
Course (month), median [IQR]	3.5 [1.0, 12.0]^‡^	2.0 [1.0, 12.0]	6.0 [2.0, 12.0]*	0.029
Glucocorticoids, n (%)	248 (64.2)	47 (72.3)	53 (64.6)	0.456
CTX, n (%)	118 (30.5)	31 (47.7)^†^	25 (30.5)*	0.025
CsA, n (%)	76 (19.6)	9 (13.8)	13 (15.9)	0.498
TAC, n (%)	152 (39.4)	24 (36.9)	27 (32.9)	0.552

IMN group: IMN without FSGS group; tpFSGS group: IMN with tip variant FSGS group; ntpFSGS group: IMN with non-tip variant FSGS group. eGFR, estimated glomerular filtration rate; MBP, mean blood pressure; TC, serum total cholesterol; TG, triglyceride; HDL, high-density lipoprotein; LDL, low-density lipoprotein. CTX, cyclophosphamide; CsA, cyclosporine A; TAC, Tacrolimus.

^†^ *p* < 0.05, IMN group vs. tpFSGS group.

^‡^ *p* < 0.05, IMN group vs. ntpFSGS group.

* *p* < 0.05, tpFSGS group vs. ntpFSGS group.

The positive rate of serum anti-PLA2R antibody was calculated by the number of positive cases to the total number of those tested.

### Pathological parameters

**Figure 2.**
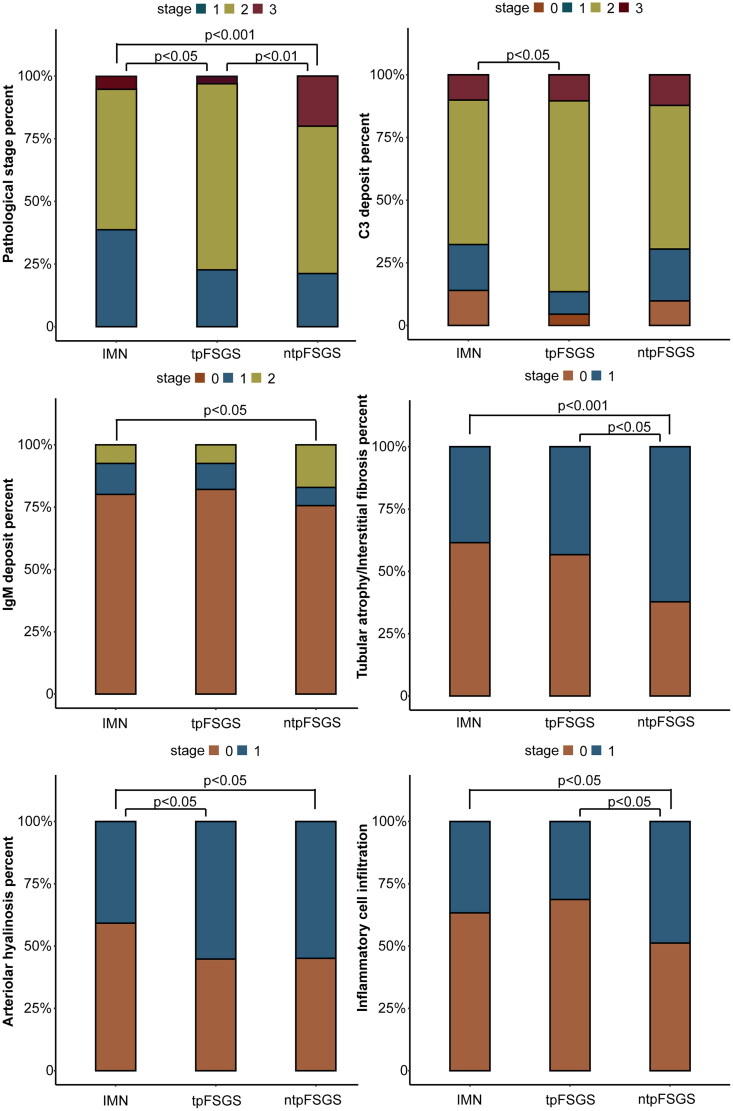
Baseline comparison of pathological parameters among groups. IMN group: IMN without FSGS group; tpFSGS group: IMN with tip variant FSGS group; ntpFSGS group: IMN with non-tip variant FSGS group.

The results are shown in Table 2 and Figure 2. Compared with the IMN group, the tpFSGS group presented a significantly greater proportion of mesangial hypercellularity (*p <* 0.05), a greater proportion of arteriolar hyalinosis (*p <* 0.05), a more advanced pathological stage (*p <* 0.05), and a greater degree of C3 staining intensity (*p <* 0.05). Compared with the IMN group, the ntpFSGS group had a significantly greater percentage of global sclerosis (*p <* 0.05), a greater proportion of mesangial hypercellularity (*p <* 0.05), a greater proportion of chronic renal tubulointerstitial lesions (*p <* 0.001), a greater proportion of arteriolar hyalinosis (*p <* 0.05), a more advanced pathological stage (*p <* 0.001), a greater proportion of interstitial inflammatory cell infiltration (*p <* 0.05), and a greater intensity of IgM staining (*p <* 0.05). Moreover, compared with the tpFSGS group, the ntpFSGS group had a significantly advanced pathological stage (*p <* 0.05), a greater percentage of global sclerosis (*p <* 0.05), a greater proportion of chronic renal tubulointerstitial lesions (*p <* 0.001), and a greater proportion of interstitial inflammatory cell infiltration (*p <* 0.05).

**Table 2.** Comparison of the pathological manifestations among three groups at the time of renal biopsy.

	IMN group	tpFSGS group	ntpFSGS group	*p*
n	387	67	82	
Global sclerosis (%), median [IQR]	4.35 [0.00, 10.76]^‡^	3.57 [0.00, 8.51]	6.67 [0.00, 14.68]*	0.022
Mesangial hypercellularity, n (%)	153 (39.5)^‡^	40 (59.7)^†^	44 (53.7)	0.002
benign arteriolar nephrosclerosis, n (%)	71 (18.3)	20 (29.9)^†^	21 (25.6)*	0.053
IgG deposit, n (%)				0.170
1	1 (0.3)	1 (1.5)	1 (1.2)	
2	45 (11.6)	4 (6.0)	8 (9.8)	
3	317 (81.9)	54 (80.6)	63 (76.8)	
4	24 (6.2)	8 (11.9)	10 (12.2)	
IgM deposit, n (%)	^‡^			0.062
0	310 (80.1)	55 (82.1)	62 (75.6)	
1	48 (12.4)	7 (10.4)	6 (7.3)	
2	29 (7.5)	5 (7.5)	14 (17.1)	
IgA deposit, n (%)	57 (14.7)	15 (22.4)	13 (15.9)	0.285
C3 deposit, n (%)		^†^		0.067
0	54 (14.0)	3 (4.5)	8 (9.8)	
1	71 (18.3)	6 (9.0)	17 (20.7)	
2	223 (57.6)	51 (76.1)	47 (57.3)	
3	39 (10.1)	7 (10.4)	10 (12.2)	
C4 deposit, n (%)	22 (5.7)	2 (3.0)	7 (8.5)	0.348
C1q deposit, n (%)	36 (9.3)	2 (3.0)	6 (7.3)	0.209
Fibrinogen deposit, n (%)	27 (7.0)	6 (9.0)	7 (8.5)	0.785

IMN group: IMN without FSGS group; tpFSGS group: IMN with tip variant FSGS group; ntpFSGS group: IMN with non-tip variant FSGS group.

^†^ *p* < 0.05, IMN group vs. tpFSGS group.

^‡^ *p* < 0.05, IMN group vs. ntpFSGS group.

* *p* < 0.05, tpFSGS group vs. ntpFSGS group.

### Prognosis and risk factors

**Figure 3.**
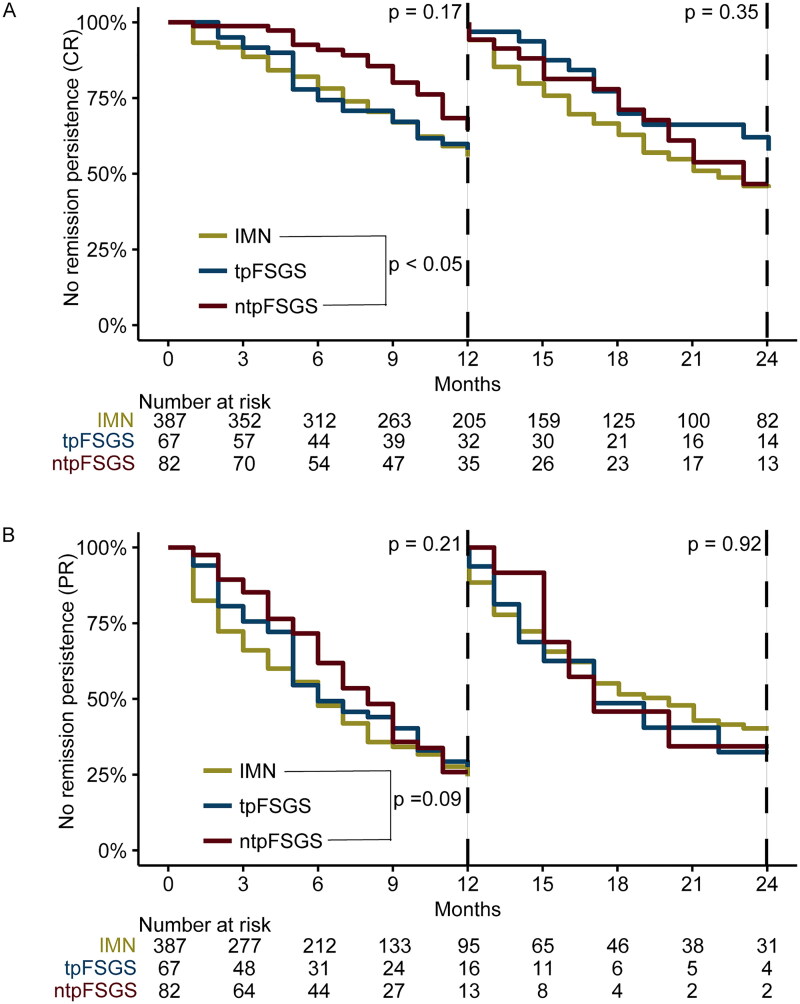
KM-Curve between different groups according to different endpoint event. (a) The endpoint event was complete remission, defined as a urine protein-to-creatinine ratio of less than 300 mg/g accompanied by normal renal function and serum albumin levels exceeding 35 g/L. IMN group had a significantly better prognosis than IMN with non-tip variant FSGS group when the follow-up endpoint was cut off at 1 year (*p* < 0.05). (b) The endpoint event was partial remission, defined as urine protein-to-creatinine ratio less than 3500 mg/g and a reduction of over 50% from the baseline, in addition to stable renal function. The comparison of prognosis at 1 year and beyond did not exhibit any significant differences among the groups. IMN group: IMN without FSGS group; tpFSGS group: IMN with tip variant FSGS group; ntpFSGS group: IMN with non-tip variant FSGS group.

A total of 144 patients did not achieve CR; this group of patients comprised 79 of 387 patients in the IMN group, 24 of 67 patients in the tpFSGS group, and 41 of 82 patients in the ntpFSGS group. Among patients who achieved CR, the median duration of remission was 11.5 months in the IMN group, 10 months in the tpFSGS group, and 13 months in the ntpFSGS group. When the endpoint of CR was considered, the KM curve (Figure 3a) revealed a significant difference in remission between the IMN group and the ntpFSGS group, with a cutoff point of 1 year (*p <* 0.05), whereas there was no significant difference in the prognosis between the tpFSGS group and the IMN group (*p* = 0.74). When the endpoint of PR was considered, the KM curve (Figure 3b) revealed no obvious differences in remission among the three groups.

Risk factors contributing to the inability to achieve CR were investigated through Cox regression analysis (Table 3). Univariate Cox regression analysis revealed that the presence of ntpFSGS (*p <* 0.05), increased age (*p <* 0.001), increased urinary protein (*p <* 0.001), increased serum fibrinogen (*p <* 0.05), and increased serum urea (*p <* 0.05) were risk factors, whereas increased serum albumin (*p <* 0.001) and increased eGFR (*p <* 0.001) were protective factors. Multivariate Cox regression analysis revealed that the presence of tpFSGS (*p <* 0.05), the presence of ntpFSGS (*p <* 0.05), increased protein (*p <* 0.001), and increased age (*p <* 0.001) were risk factors. Moreover, compared with the use of renin-angiotensin system inhibitors (RASIs), the use of glucocorticoids with cyclophosphamide (*p <* 0.001) was a protective factor in the multivariate Cox analysis.

**Table 3.** Factors associated with no remission.

Variables	Univariate Cox regression			Multivariate Cox regression		
	HR	CI	*p*	HR	CI	*p*
Age (year)	0.98	0.97–0.99	0.000	0.98	0.97–0.99	<0.001
Urinary Protein (g/24 h)	0.94	0.91–0.97	0.000	0.94	0.9–0.97	<0.001
FSGS with tip lesion	0.80	0.57–1.11	0.173	0.69	0.49–0.96	0.029
FSGS with non-tip lesion	0.71	0.51–0.99	0.043	0.66	0.47–0.92	0.014
CNI+/-GCs	1.00	0.77–1.3	0.977	1.06	0.81–1.38	0.657
CTX+GCs	1.83	1.37–2.45	0.000	2.24	1.65–3.04	<0.001
Other treatment plans	0.59	0.42–0.84	0.003	0.64	0.44–0.92	0.016
eGFR (mL/min/1.73 m^2^)	1.01	1.01–1.02	0.000			
Fibrinogen deposit	1.02	0.71–1.49	0.898			
Global glomerulosclerosis (%)	0.99	0.98–1	0.060			
Serum albumin (g/L)	1.08	1.06–1.1	0.000			
Serum urea (mmol/L)	0.93	0.87–0.99	0.018			
Serum fibrinogen (g/L)	0.90	0.83–0.97	0.007			
Serum uric acid (mmol/L)	1.00	1–1	0.262			
Female	1.09	0.89–1.34	0.383			
Stage of membranous nephropathy	0.86	0.72–1.03	0.097			
Tubular atrophy/Interstitial fibrosis	0.81	0.62–1.07	0.137			
Vascular hyaline degeneration	0.94	0.77–1.15	0.537			
IgG deposit	0.90	0.72–1.12	0.346			
Mesangial proliferative	1.14	0.93–1.39	0.203			

eGFR, estimated glomerular filtration rate; LDL, low-density lipoprotein; HDL, high-density lipoprotein. The treatment plans were divided into the following groups as a categorical variable: 1. RASI: Patients only use angiotensin-converting enzyme inhibitor (ACEI) or angiotensin receptor blocker (ARB) within 2 years of onset; (This group as a control variable) 2. CNI ± GCs: On the basis of RASI, patients use calcineurin inhibitors (CNI) in combination with glucocorticoids (GCs) or use calcineurin inhibitors alone within 2 years of onset; 3. CTX+GCs: On the basis of RASI, patients use cyclophosphamide (CTX) in combination with Glucocorticoids within 2 years of onset; 4. Other treatment plans: Medication plans other than the above situations, including glucocorticoids alone, other immunosuppressants, and combined use of multiple immunosuppressants. The outcome variable is complete remission (CR).

## Discussion

Previous studies have analyzed the clinicopathological characteristics of idiopathic membranous nephropathy patients with FSGS. However, most of the studies had small sample sizes, and few of them subdivided IMN patients with FSGS into those with the tip variant and those with the non-tip variant. The aim of the present study was to investigate the impact of specific FSGS variants on the clinicopathological features and prognosis of IMN. Compared with IMN patients, IMN patients with FSGS had greater clinical manifestations of urinary protein, albumin, lipids, and blood creatinine at disease onset and a greater proportion of positive serum anti-PLA2R antibodies. Previous studies have shown that serum anti-PLA2R antibody titers are correlated with disease severity [19,20]. Analysis of the pathological features revealed that the pathological stage was more advanced and that the proportions of patients with arteriolar hyalinosis and mesangial hypercellularity were greater in both the tpFSGS and ntpFSGS groups. Light microscopic pathologic changes revealed that the presence of FSGS in IMN patients was accompanied by a more severe inflammatory response.

In patients diagnosed with IMN, the clinicopathologic manifestations of the ntpFSGS group were similar to those observed in the tpFSGS group. Nevertheless, the ntpFSGS group presented certain peculiarities. Compared with the IMN group, the ntpFSGS group had greater blood fibrinogen, which was related to the inflammatory state reported in a previous study [21]. In our study, the highest proportion of chronic renal tubulointerstitial, the most advanced pathological stages, the highest proportion of interstitial inflammatory cell infiltration, and the highest proportion of global sclerosis were found in the ntpFSGS group. The worse clinicopathologic manifestations indicated that the ntpFSGS group had more advanced lesions than did the tpFSGS group. In this study, the ntpFSGS group presented increased IgM deposition, which was very similar to the nonspecific deposition of immunoglobulins shown in idiopathic FSGS [22].

On the one hand, multivariate Cox regression analysis revealed that tpFSGS was a risk factor for failure to achieve CR, but on the other hand, the KM curve revealed that there was no significant difference in remission between the IMN group and the tpFSGS group. Considering that the multivariate Cox regression analysis incorporated therapeutic factors, these two different outcomes indicated that tpFSGS may result in failure to achieve remission, but remission can be achieved after aggressive immunosuppressive therapy. In a previous study of primary FSGS, Stokes et al. reported that tpFSGS patients responded well to immunosuppressive therapy and had a benign clinical outcome [23]. Similarly, in the present study, we found that there was a trend toward better remission in the tpFSGS group, which may be due to the better response to immunosuppressive therapy, but the specific mechanism needs to be further investigated. Nevertheless, ntpFSGS was found to be a risk factor *via* multivariate Cox regression analysis for failure to achieve CR, and the KM curve revealed a significant difference in remission between the IMN group and the ntpFSGS group. The results of the two analyses indicated that the ntpFSGS group had a worse short-term prognosis.

Podocyte damage plays a key role in primary and secondary FSGS [24]. When podocytes are severely detached beyond the compensatory range, the basement membrane adheres to Bowman’s capsule, and exuded plasma proteins and matrix proteins are formed, ultimately leading to glomerulosclerosis [25–27]. PLA2R is expressed in the cytoplasm and cytosol of normal glomerular podocytes, and PLA2R is the target antigen in approximately 70% of adult IMNs [28,29]. Anti-PLA2R antibodies can lead to podocyte damage, although the mechanism is unclear [29]. In the present study, we detected a greater proportion of positive serum anti-PLA2R antibodies in IMN patients with FSGS, implying that more M-type phospholipase A2 receptors in the glomerulus were saturated and bound to anti-PLA2R, which caused more severe podocyte injury.

Mesangial cell injury can lead to segmental sclerosis. Without mesangial support, podocytes can compensate for a limited period of time to maintain normal glomerular basement membrane structure and function, but prolonged loss of mesangial support can cause the proximal tubule to insert into the glomerulus, resulting in podocyte damage and tpFSGS [30]. Moreover, mesangial cells can also mediate the loss of podocyte nephrin protein through the secretion of platelet-activating factor (PAF) [31]. In the present study, we found that mesangial hypercellularity was more obvious in IMN patients with FSGS and that mesangial hypercellularity may also be a potential mechanism for the development of FSGS in IMN patients. FSGS is associated with a reduced number of nephrons, adaptive hemodynamic changes, and changes in hyperfiltration [32]. In the present study, we found that the ntpFSGS group had a lower eGFR and a greater percentage of global sclerosis, which implied greater nephron loss. In conclusion, for IMN patients, the persistence of tip or non-tip variant FSGS shares similar mechanisms, such as podocyte and mesangial injury, and nephron loss may be an additional mechanism for the occurrence of ntpFSGS. Combining clinicopathologic features, prognostic characteristics, and potential pathogenesis, the tip variant and non-tip variant are likely different stages of secondary FSGS lesions, with the tip variant type being in the early stage of FSGS, and the tip variant possibly progresses to a non-tip variant under the natural course of IMN, which is worthy of further study.

Our study revealed that both tpFSGS and ntpFSGS demonstrated a significantly higher prevalence of arteriolar hyalinosis compared to IMN. Notably, a greater proportion of patients in these groups exhibited concurrent benign arteriolar nephrosclerosis. It is well-established that hypertension directly contributes to arteriolar hyalinosis through sustained vascular endothelial injury and subintimal protein deposition. Recent investigations demonstrate synergistic associations between metabolic disorders (particularly obesity), smoking exposure, and the development of arteriolar hyaline lesions [33,34]. The elevated incidence of arteriolar hyalinosis in tpFSGS and ntpFSGS cohorts implies potential etiological links among hypertension, smoking, obesity, and FSGS development. These comorbidities collectively induce glomerular hyperfiltration, podocyte stress, and subsequent FSGS lesion formation. Moreover, clinical investigations have consistently identified hypertension, smoking, and obesity as independent predictors of unfavorable renal outcomes [35,36]. Future studies are warranted to investigate the prognostic implications of these comorbidities in MN patients complicated with FSGS lesions.

## Conclusions

In conclusion, IMN patients with FSGS had more severe clinical manifestations and worse renal function. IMN patients with non-tip variant FSGS had significantly more severe tubulointerstitial lesions, more severe interstitial inflammatory cell infiltration, and lower CR. Tip variant FSGS is also an independent risk factor for failure to achieve CR in IMN patients, but after aggressive immunosuppressive therapy, CR is not different from that of IMN patients without FSGS. Most likely, tip and non-tip variant FSGS are different stages of FSGS in IMN.

## Supplementary Material

Supplemental Material

## Data Availability

Due to ethical considerations, the data used in this study are not publicly available. For any further inquiries regarding the data, interested individuals can contact the corresponding author.
