# Supplementary material for: Clinicopathological and prognostic study of idiopathic membranous nephropathy with tip or non-tip variant focal segmental glomerulosclerosis: a single-center cohort study
Source: Ren Fail. 2025 Jun 8;47(1):2512052. doi: 10.1080/0886022X.2025.2512052 (PMC12147480; doi:10.1080/0886022X.2025.2512052)
Supplement: Supplemental Material [file IRNF_A_2512052_SM3716.docx]

| Supplementary S1. Post-hoc analysis of clinical manifestations among three groups | | | |
| --- | --- | --- | --- |
| Parameters | IMN vs. tpFSGS | IMN vs. ntpFSGS | tpFSGS vs. ntpFSGS |
| Age (year) | 0.708 | 0.355 | 0.702 |
| eGFR (mL/min/1.73m2) | 0.136 | 0.016 | 0.549 |
| MBP (mmHg) | 0.185 | 0.349 | 0.939 |
| Serum creatinine (umol/l) | 0.049 | 0.035 | 0.997 |
| Proteinuria (g/24h) | 0.023 | 0.021 | 0.895 |
| Serum albumin (g/L) | 0.002 | 0.000 | 0.662 |
| TC (mmol/L) | 0.088 | 0.003 | 0.415 |
| TG (mmol/L) | 0.036 | 0.214 | 0.448 |
| HDL (mmol/L) | 0.003 | 0.005 | 0.739 |
| LDL (mmol/L) | 0.277 | 0.003 | 0.173 |
| UA (mmol/L) | 0.431 | 0.361 | 0.191 |
| Hemoglobin (g/L) | 0.439 | 0.259 | 0.145 |
| Serum fibrinogen (g/L) | 0.287 | 0.004 | 0.205 |
| Blood urea (mmol/L) | 0.137 | 0.253 | 0.730 |

| Supplementary S2. Comparison of therapeutic regimen among groups in two years | | | | |
| --- | --- | --- | --- | --- |
|  | IMN | tpFSGS group | ntpFSGS group | *P* |
| n | 387 | 67 | 82 |  |
| Whether to use | | | | |
| Glucocorticoids (%) | 248 (64.2) | 47 (72.3) | 53 (64.6) | 0.456 |
| CTX (%) | 118 (30.5) | 31 (47.7) ^†^ | 25 (30.5) * | 0.025 |
| CsA (%) | 76 (19.6) | 9 (13.8) | 13 (15.9) | 0.498 |
| TAC (%) | 152 (39.4) | 24 (36.9) | 27 (32.9) | 0.552 |
| ACEI/ARB (%) | 350 (90.4) ^‡^ | 55 (82.1) | 64 (78.0) | 0.004 |
| Total dose | | | | |
| Glucocorticoids (mg), median [IQR] | 1445.0 [0.0, 3500.0] | 2000.0 [0.0, 3650.0] | 1047.5 [0.0, 3125.0] | 0.251 |
| CTX (mg),  median [IQR] | 0.0 [0.0, 1200] | 0.0 [0.0, 3400] ^†^ | 0.0 [0.0, 1000] * | 0.055 |
| CsA (mg),  median [IQR] | 0.0 [0.0, 0.0] | 0.0 [0.0, 0.0] | 0.0 [0.0, 0.0] | 0.428 |
| TAC (mg),  median [IQR] | 0.0 [0.0, 300.0] | 0.0 [0.0, 102.0] | 0.0 [0.0, 142.5] | 0.476 |
| Total dose in patients using medication (mg) | | | | |
| Glucocorticoids (mg), median [IQR] | 2775.0  [1500.0, 4500.0] | 3000.0  [1957.5, 4600.0] | 2455.0  [1230.0, 4600.0] | 0.362 |
| CTX (mg),  median [IQR] | 4200 [1800, 6500] | 3600 [2400, 4800] | 3600 [1800, 6400] | 0.403 |
| CsA (mg),  median [IQR] | 22500.0  [7450.0, 45262.5] | 21200.0  [4600.0, 33750.0] | 27300.0  [10600.0, 35000.0] | 0.854 |
| TAC (mg),  median [IQR] | 411.0 [171.2, 700.0] | 358.0 [71.8, 800.0] | 353.0 [150.0, 627.5] | 0.804 |
| Duration of medication in patients using medication (month) | | | | |
| Glucocorticoids (month), mean (SD) | 7.4 (4.5) | 7.6 (5.2) | 6.6 (4.9) | 0.465 |
| CTX (month),  mean (SD) | 6.0 (3.8) | 5.8 (3.7) | 5.3 (3.9) | 0.710 |
| CsA (month),  mean (SD) | 9.1 (6.6) | 8.4 (6.7) | 9.9 (7.2) | 0.867 |
| TAC (month),  mean (SD) | 8.4 (6.0) | 7.8 (7.1) | 8.0 (6.4) | 0.887 |
| IMN group: IMN without FSGS group; tpFSGS group: IMN with tip variant FSGS group; ntpFSGS group: IMN with non-tip variant FSGS group. †P<0.05, IMN group vs. tpFSGS group; ‡P<0.05, IMN group vs. ntpFSGS group; *P<0.05, tpFSGS group vs. ntpFSGS group. ACEI /ARB, angiotensin-converting enzyme inhibitor or angiotensin receptor blocker; CTX, cyclophosphamide; CsA, cyclosporine A; TAC, Tacrolimus. Statistic of glucocorticoids dose is transformed into equivalent dose of prednisone. | | | | |

| Supplementary S3. Partial remission rate comparison by different treatment among three groups | | | |
| --- | --- | --- | --- |
| Treatment | IMN group | tpFSGS group | ntpFSGS group |
| One-year remission rate |  |  |  |
| ACEI/ARB | 69% (66/96) ^‡^ | 55% (6/11) | 32% (6/19) |
| CTX+GCs | 94% (66/70) ^‡^ | 77% (17/22) ^†^ | 61% (11/18) |
| CNI+/-GCs | 80% (98/123) | 67% (12/18) | 79% (22/28) |
| Other treatment | 57% (56/98) | 44% (7/16) | 41% (7/17) |
| Two-year remission rate |  |  |  |
| ACEI/ARB | 79% (76/96) ^‡^ | 73% (8/11) | 37% (7/19) * |
| CTX+GCs | 99% (69/70) ^‡^ | 82% (18/22) ^†^ | 72% (13/18) |
| CNI+/-GCs | 90% (111/123) | 78% (14/18) | 82% (23/28) |
| Other treatment | 73% (72/98) | 69% (11/16) | 53% (9/17) |
| IMN group: IMN without FSGS group; tpFSGS group: IMN with tip variant FSGS group; ntpFSGS group: IMN with non-tip variant FSGS group. ^†^P<0.05, IMN group vs. tpFSGS group; ^‡^P<0.05, IMN group vs. ntpFSGS group; *P<0.05, tpFSGS group vs. ntpFSGS group. Definition of different treatment options: 1. RASI: Patients only use angiotensin-converting enzyme inhibitor (ACEI) or angiotensin receptor blocker (ARB) within two years of onset; 2. CNI+/-GCs: On the basis of RASI, patients use calcineurin inhibitors (CNI) in combination with glucocorticoids (GCs) or use calcineurin inhibitors alone within two years of onset; 3. CTX+GCs: On the basis of RASI, patients use cyclophosphamide (CTX) in combination with Glucocorticoids within two years of onset; 4.other treatment plans: Medication plans other than the above situations, including glucocorticoids alone, other immunosuppressants and combined use of multiple immunosuppressants. | | | |

| Supplementary S4. Test for Proportional hazards | | | |
| --- | --- | --- | --- |
| Variables | Model1 |  | Model2 |
|  | *P* |  | *P* |
| Age | 0.014 |  | 0.089 |
| Urinary Protein | 0.618 |  | 0.650 |
| Group | 0.177 |  | 0.163 |
| Treatment | 0.341 |  | 0.328 |
| Global | 0.067 |  | 0.150 |
| Group (categorical variable): IMN (as a control variable), tpFSGS and ntpFSGS. Treatment (categorical variable): RASI (as a control variable), CNI+/-GCs, CTX+GCs and Other treatment plans. Model1: multivariate Cox regression model without time stratification. Model2: divide the analysis time into multiple intervals and perform Cox proportional model stratification on these time intervals. | | | |

| Supplementary S5. Multivariate Cox regression with interactive stratification | | | |
| --- | --- | --- | --- |
| Variables | HR |  | *P* |
| Age: strata1 | 0.97 |  | <0.001 |
| Age: strata2 | 0.98 |  | 0.014 |
| Urinary Protein | 0.94 |  | <0.001 |
| FSGS with tip lesion | 0.70 |  | 0.035 |
| FSGS with non-tip lesion | 0.66 |  | 0.016 |
| CNI+/-GCs | 1.06 |  | 0.667 |
| CTX+GCs | 2.24 |  | <0.001 |
| Other treatment plans | 0.65 |  | 0.019 |
| The covariate age showed significant time-dependent effects (Schoenfeld test p < 0.05). A stratified Cox model with time-interval partitioning was established, where age was interactively adjusted within discrete time strata. | | | |

Figure S1. The flow chart of inclusion and exclusion criteria.


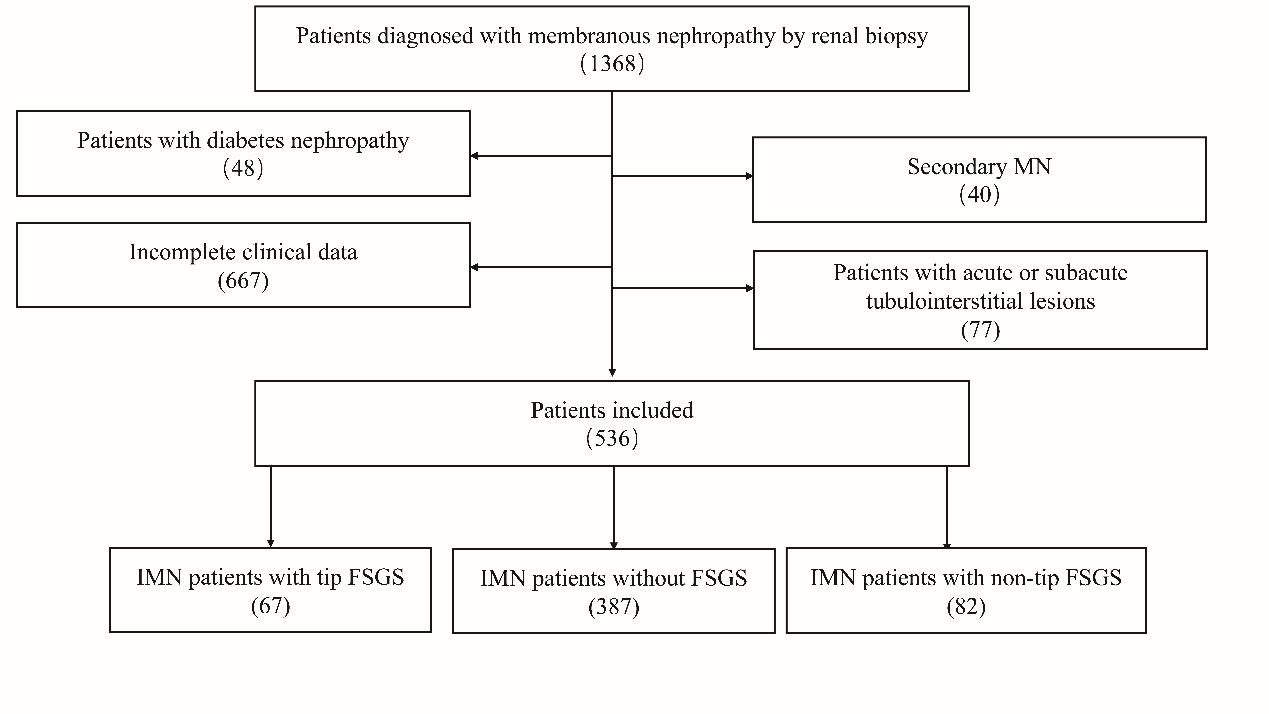


Figure S2. KM-curve between different groups according to different endpoint event


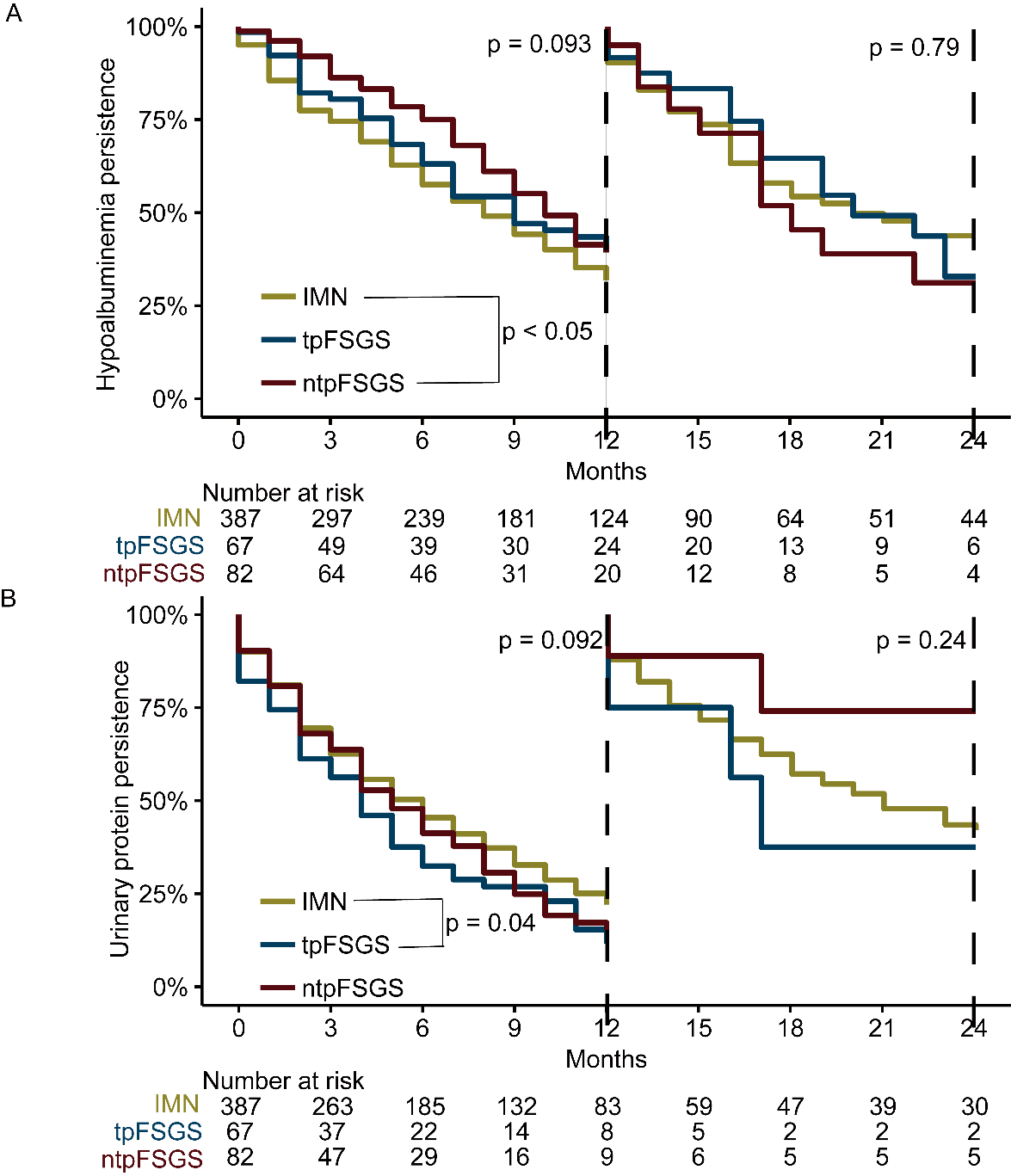


IMN group: IMN without FSGS group; tpFSGS group: IMN with tip variant FSGS group; ntpFSGS group: IMN with non-tip variant FSGS group. Figure S2A. The endpoint event was defined as an increase in albumin to 35g/L. IMN group had a significantly better prognosis than IMN with non-tip variant FSGS group when the follow-up endpoint was cut off at 1 year(P<0.05). Figure S2B.The endpoint event was defined as urinary protein-to-creatinine ratio below to 3.5g/g and below half the peak of urinary protein-to-creatinine. IMN with tip variant FSGS group had a significantly better prognosis than IMN group when the follow-up endpoint was cut off at 1 year(P=0.04).

Figure S3. KM-curve between different groups with a 50% decrease in eGFR as endpoint event


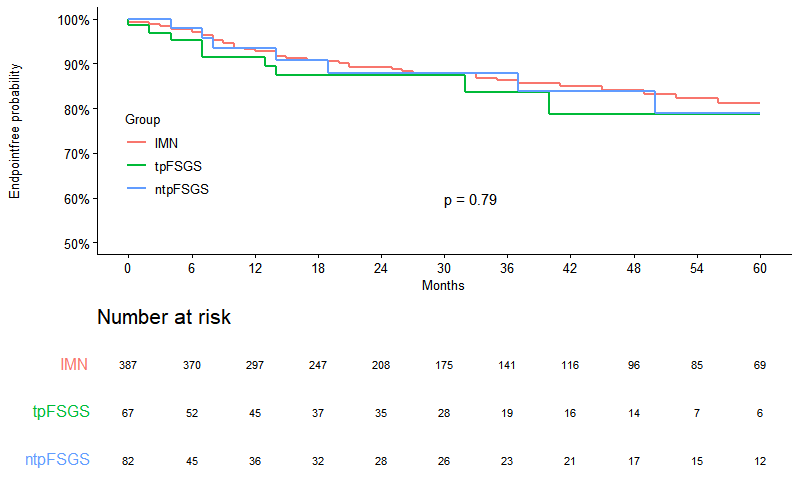
 IMN group: IMN without FSGS group; tpFSGS group: IMN with tip variant FSGS group; ntpFSGS group: IMN with non-tip variant FSGS group. The endpoint event was defined as a 50% decrease in eGFR. (P=0.79)
